# Evaluation and Optimization of Thermoplastic Extrusion Parameters Influencing the Impact Resistance of Additively Manufactured Samples from PETG and Recycled PETG

**DOI:** 10.3390/polym17182533

**Published:** 2025-09-19

**Authors:** Dragos Gabriel Zisopol, Mihail Minescu, Dragos Valentin Iacob

**Affiliations:** 1 Mechanical Engineering Department, Petroleum-Gas University of Ploiesti, 100680 Ploiesti, Romania; zisopold@upg-ploiesti.ro (D.G.Z.); mminescu@upg-ploiesti.ro (M.M.); 2 Department of Mechanical Engineering, Doctoral School, Petroleum-Gas University of Ploiesti, 100680 Ploiesti, Romania

**Keywords:** additive manufacturing, FDM, process parameters, Charpy impact test, PETG, recycled PETG, circular economy

## Abstract

Given the widespread use of additive manufacturing technologies through plastic extrusion and the need to use recycled plastic materials, this paper presents the results of the study on the evaluation and optimization of the influence of theromoplastic extrusion parameters on the impact resistance of additively manufactured samples from PETG and recycled PETG (rPETG) filament from the Everfil brand. In this context, 90 impact samples, 45 from PETG and 45 from rPETG, were additively manufactured by thermoplastic extrusion by the QIDI Q1 Pro printer, with the layer height deposited per pass L_h_ = 0.10/0.15/0.20 mm and the filling percentage I_d_ = 50/75/100%, which were subsequently subjected to impact testing by the HST XJJD-50T machine, using the 7.5J hammer and the impact speed of 2.9 m/s. In order to statistically evaluate the influence of the variable parameters of thermoplastic extrusion, layer height per pass (L_h_) and filling percentage (I_d_), on the impact strengths of additively manufactured PETG and rPETG samples, ANOVA and DOE analyses were performed using Minitab 20.3 software. Using the determined optimal parameters (L_h_ = 0.10 mm and I_d_ = 100%), impact strength values were obtained that were 210.87% higher than the impact strength values obtained from testing PETG samples. Considering the impact strength results obtained for the samples manufactured from rPETG and the fact that rPETG filament is 11% cheaper per kilogram than PETG filament, it can be concluded that the use of rPETG filament is a viable solution for the additive manufacturing of parts by thermoplastic extrusion.

## 1. Introduction

The increase in plastic production has generated the global problem of efficient waste management. The shift to a circular economy-based production model is a necessity, and postponing the adoption of the circular economy in the plastics sector not only generates negative effects on ecosystems but also on the smooth running of daily activities [1,2,3,4,5,6,7,8]. PET (polyethylene terephthalate) is a thermoplastic polymer with high mechanical strength, good ductility and processability, which makes it ideal for applications in the medical industry, the cosmetics industry and the packaging industry [9,10,11,12,13,14,15,16]. The upward evolution of the use of PET and PETG (polyethylene terephthalate glycol) in additive manufacturing processes by thermoplastic extrusion has generated the need to research the mechanical performance of these materials in the context of a sustainable use [17,18,19,20,21,22,23,24,25,26,27,28,29,30,31,32]. To maximize the mechanical and quality characteristics, it is necessary to optimize the thermoplastic extrusion parameters used in the manufacture of parts. In this context, in paper [33], a study was carried out on the optimization of thermoplastic extrusion parameters with the aim of improving the mechanical properties in tension, bending and impact. The following printing parameters were analyzed in the study: layer height, filling percentage, raster angle, printing speed and extrusion temperature. The conclusions of the study highlight the need to optimize manufacturing parameters to obtain superior mechanical characteristics. In paper [34], the authors analyzed the relationship between thermoplastic extrusion parameters (extrusion temperature, printing speed, layer height and cooling rate) and 3D printed parts defects (cooling deformation, under-extrusion, excessive porosity and poor adhesion between layers). To optimize the parameters, the authors used Taguchi, ANOVA and RSM type analyses. The obtained results confirm that the optimal extrusion temperature varies depending on the 3D-printed material and that it is recommended to be (5–10) °C above the melting point of the material. To reduce defects caused by printing speed, it is recommended to set it in the range (40–60) mm/s. The study carried out in paper [35] highlights the influence of thermoplastic extrusion parameters on mechanical properties in tension, compression, bending and impact, analyzing the results of over 100 experimental tests. The optimal values of extrusion temperatures for PLA are in the range (200–220) °C and (230–250) °C for ABS, with the increase in extrusion temperature ensuring the improved adhesion of the layers. The optimal values of 3D printer platform temperatures are in the range (60–70) °C for PLA and (100–110) °C for ABS, with the increase in platform temperature ensuring good bonding of the first deposited layer and a low probability of warping defects. The height of the layer is recommended to be in the range (0.10–0.30) mm, as decreasing the height of the layer generates an increase in the mechanical resistance of the 3D-printed part and the printing time. The recommended printing speeds are in the range (40–80) mm/s, as speeds higher than 100 mm/s lead to a decrease in the mechanical resistance of the 3D-printed parts due to the occurrence of the under-extrusion defect. To obtain superior mechanical characteristics, it is recommended to set the filling percentage of 3D-printed parts in the range (80–100)%. The study presented in paper [36] highlights the importance of choosing the appropriate material and optimizing the thermoplastic extrusion parameters depending on the field of use of 3D-printed parts. PLA is a cheap and easily printable material; however, composite materials (PLA-CF) or PEEK (polyetheretherketone) are recommended for industrial applications. In paper [37], the parametric optimization of the FDM process for 3D-manufactured parts from PA12-CF is presented, using the response surface methodology (RSM), grey relational analysis (GRA) and grey wolf optimization (GWO). The analyzed parameters were extrusion temperature (240–280) °C; printing speed (30–70) mm/s; speed of the deposited layer (0.10–0.30) mm; filling percentage (50–100)%. As a result of the optimization, the following values were obtained: extrusion temperature 265 °C; printing speed 45 mm/s; height of the deposited layer 0.15 mm; filling percentage 90%. In work [38], the effect of process parameters and material selection on the quality of parts manufactured by thermoplastic extrusion was analyzed. The conclusions of the study provide recommendations regarding the choice of materials. PLA is recommended for 3D manufacturing of parts subjected to low mechanical stress. So is PETG/ABS, with the specification that they confer better mechanical properties to 3D-printed parts. Composite materials are recommended for 3D manufacturing of parts subjected to high mechanical stress. Optimization of manufacturing parameters is essential for obtaining the mechanical characteristics required for 3D-printed parts. In article [39], the authors present the stages of the study and the results obtained from optimizing the parameters of thermoplastic PLA extrusion (using the methods Taguchi, linear and nonlinear regression and artificial neural networks) in order to reduce defects in 3D-extruded parts and maximize their mechanical strength. The following parameters were analyzed: extrusion temperature (190–230) °C; printing speed (30–90) mm/s; deposited layer height (0.10–0.30) mm; filling percentage (20–100)%. The Taguchi method highlighted the filling percentage and extrusion temperature as the main parameters that significantly influenced the tensile strengths of 3D-printed parts. The regression modeling was validated experimentally, with an average error of ±3%. The accuracy of using neural networks (ANNs) was 98% in predicting the tensile strength. After optimization, the following values were obtained: extrusion temperature 200 °C; printing speed 50 mm/s; layer height deposited per pass 0.20 mm; filling percentage 80%. In [40], the authors optimized the 3D printing parameters of tensile, bending and impact samples made of PA6 with a PA6GF30 core. The solution of using the two materials in successive layers reduced the delamination phenomenon and gave the PA6 samples with a PA6GF30 core improved mechanical resistance compared with those made of PA6 or PA6GF30.

The implementation of the use of recycled materials in the field of additive manufacturing technologies is a topical topic that offers numerous research directions. In the context of this article and of the global trend towards a circular economy, a bibliographic study was conducted on the current state of research in the field of additive manufacturing technology through thermoplastic extrusion of recycled PET and PETG materials. In paper [41], the authors present the results obtained from the combination of recycled polyethylene terephthalate (rPET) with pyromellitic dianhydride (PMDA). The combination of rPET with PMDA led to improved processability and mechanical performance of 3D-printed parts, which are comparable to those obtained for virgin materials. The study presented in paper [42] refers to the influence of high-density polyethylene (HDPE) contamination of recycled polyethylene terephthalate (rPET) and highlights the need for efficient sorting of plastic waste. For the study, rPET filament with a controlled content of (0–10)% HDPE was manufactured. After determining the mechanical properties of tensile, bending and impact of 3D-printed samples with the filament resulting from the combination of rPET with HDPE, the major influence of HDPE contamination on the mechanical properties of rPET was demonstrated. The high HDPE content caused the appearance of major defects in the 3D-printed parts and significant decreases in their mechanical properties.

In [43], a study on the optimization of thermoplastic extrusion parameters of PETG and recycled PETG (rPETG) for the manufacture of samples used in the three-point bending test is presented in the context of the transition to a circular economy. The study was carried out using 90 samples for the three-point bending test (45 from PETG and 45 from rPETG), 3D printed by thermoplastic extrusion. The results of the study confirmed that the variable parameters of 3D printing by thermoplastic extrusion, the height of the deposited layer (L_h_) and the filling percentage (I_d_), influenced the simple bending behavior of samples manufactured from PETG and rPETG. The average bending strength results of PETG samples were 9.19% higher than the bending results of rPETG samples. In paper [44], the results obtained from mechanical tests performed on 3D samples manufactured by thermoplastic extrusion of PETG and the same material recycled several times are presented. Repeated recycling of PETG decreased the mechanical characteristics of the material: tensile strength by 15%, bending strength by 12%, impact strength by 30% and hardness by 5%. The article concludes that PETG can be recycled 4/5 times with acceptable losses of mechanical characteristics. In paper [45], the proportion in which recycled PETG can be used together with virgin PETG in the manufacture of filaments used in additive manufacturing is studied, without significantly affecting the mechanical properties of 3D-printed parts by thermoplastic extrusion. For the study, filaments were manufactured from 80% PETG + 20% rPETG and 60% PETG + 40% rPETG. Using these filaments, tensile, bending and impact samples were 3D printed by thermoplastic extrusion. The results obtained from mechanical tests performed on the samples demonstrated that up to 40% rPETG can be used in the manufacture of the filament, without significantly affecting the mechanical properties of the 3D-printed parts by thermoplastic extrusion.

Impact strength is of major importance in evaluating the behavior of parts subjected to dynamic stresses or those intended for use in variable conditions.

Considering the existing works in the specialized literature, research opportunities have been identified that can enrich the level of knowledge. In this regard, the objective of this work is to evaluate the influence of 3D printing parameters by thermoplastic extrusion, the height of the layer deposited in a single pass (L_h_) and the filling percentage (I_d_), on the impact resistance of samples additively manufactured from PETG and recycled PETG filament (rPETG). The novelty of this work consists in identifying the correlation between the process parameters and the impact resistance, leading to the stability of optimal values for the two parameters considered, in order to maximize the impact resistance of samples made from PETG and rPETG and to provide insights into the use of recycled materials in additive manufacturing technologies.

## 2. Materials and Experimental Procedure

### 2.1. Research Methodology

**Figure 1:**
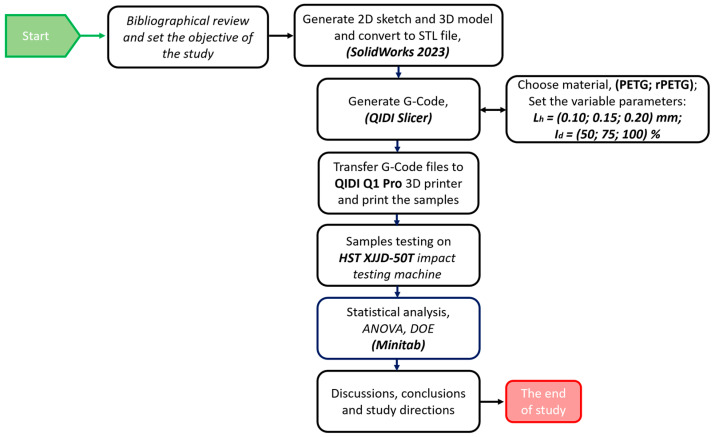
Schematic representation of the methodology employed to assess the influence of thermoplastic extrusion parameters on the impact resistance of additively manufactured PETG and rPETG samples.

In order to conduct this study evaluating the impact of thermoplastic thermoplastic extrusion parameters on the impact resistance of samples made from PETG and recycled PETG, the methodology utilized is outlined in Figure 1.

### 2.2. Bibliographical Study

**Figure 2:**
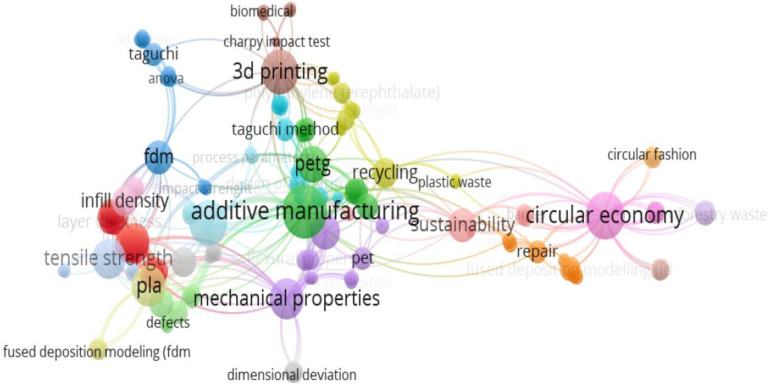
Co-occurrence network of keywords from the studied specialized literature.

Figure 2 shows the bibliometric analysis map of the co-occurrence of keywords in the studied scientific articles generated by VOS Viewer 1.6.20 software [46]. This network-like map allows visualization of the connections between keywords in the specialty literature and highlights research trends.

### 2.3. Manufacturing of Samples for Charpy Impact Testing

**Figure 3:**
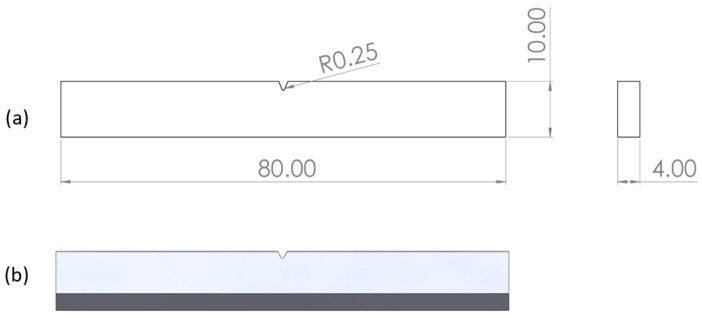
Impact testing sample in Solidworks 2023: (**a**) 2D sketch; (**b**) 3D model.

Using the ISO 179-1:2023 standard, which specifies the method for determining the Charpy impact properties of plastic materials, and Solidworks 2023 SP1 software, a 2D sketch of the specimen was made, and subsequently its 3D model was created (Figure 3) [47].

The SLDPRT file, corresponding to the 3D model of the impact sample, was converted to STL (standard triangle language) format in order to be used in the slicer of the QIDI Q1 Pro 3D printer.

**Figure 4:**
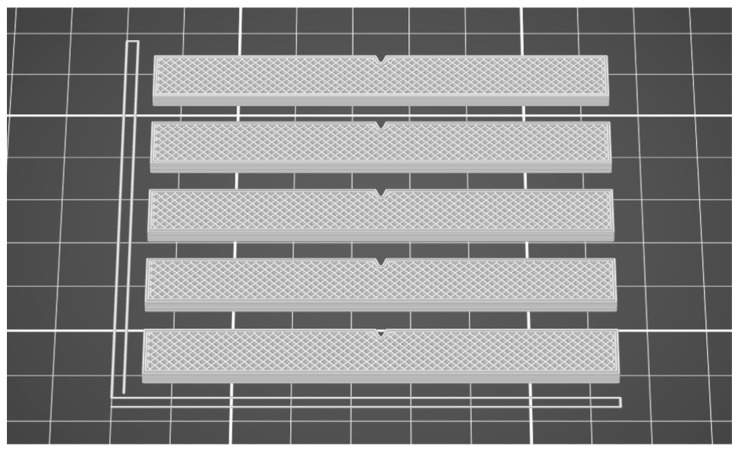
Impact testing samples in QIDISlicer.

The STL file was processed in the QIDISlicer software, version 1.2.3., by setting the process parameters according to the data presented in Table 1. Figure 4 shows the impact samples in the QIDISlicer software [48].

Using the QIDISlicer software and the parameters presented in Table 1, the G-Code files contain the work instructions for the additive manufacturing of impact samples by thermoplastic extrusion of PETG and rPETG filaments.

The G-Code files were transmitted to the QIDI Q1 Pro 3D printer (Hangzhou, Zhejiang, China), where 90 impact samples, 45 from PETG and 45 from rPETG, were manufactured by thermoplastic extrusion.

**Figure 5:**
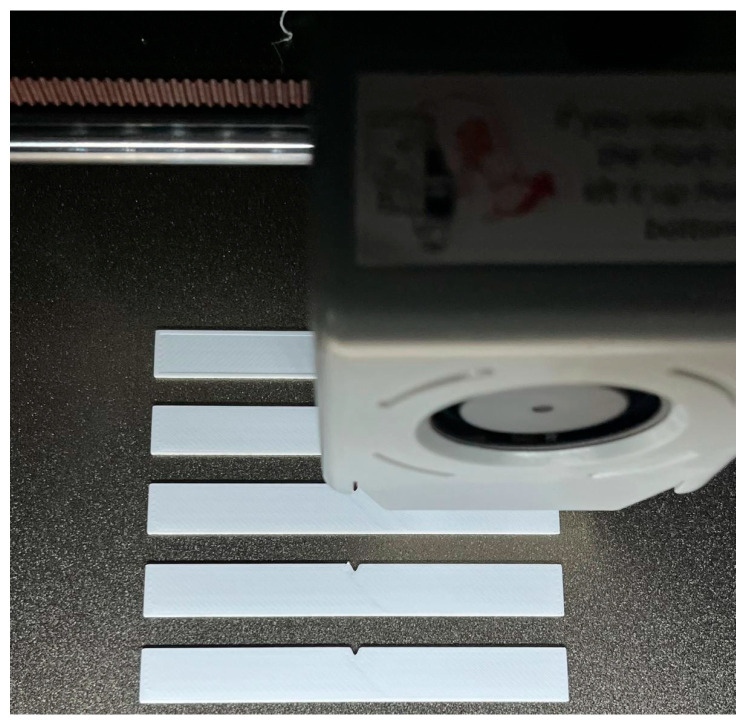
Thermoplastic extrusion of impact samples on QIDI Q1 Pro.

PETG and rPETG Everfil filament (Białystok, Poland), with a diameter of 1.75 mm was used to manufacture the samples. Accoding to the producer [49], the filament has the following physical properties: density 1.29 g/cm^2^ according to ASTM D792, [50], tensile modulus 2200 MPa according to ISO 527, [51], flexural modulus 79 MPa according to ISO 178, [52] and glass transition temperature 80 °C according ASTM D3418, [53]. Figure 5 shows the fabrication of impact samples using the QIDI Q1 Pro 3D printer.

**Figure 6:**
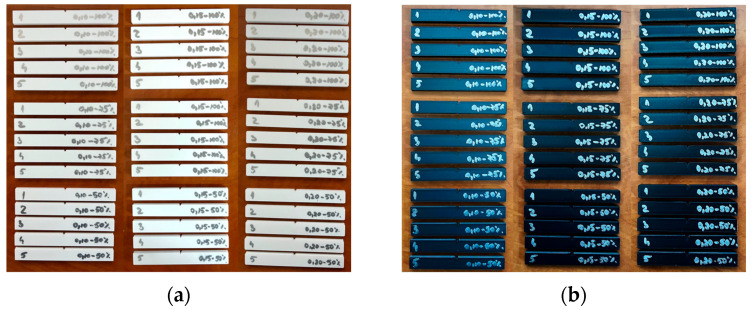
Impact test samples fabricated through additive manufacturing using the QIDI Q1 Pro 3D printer: (**a**) PETG; (**b**) rPETG.

Figure 6 shows the samples made of PETG and rPETG before impact tests.

### 2.4. Charpy Impact Testing of Samples Manufactured by Additively Thermoplastic Extrusion of PETG and rPETG Filaments

**Figure 7:**
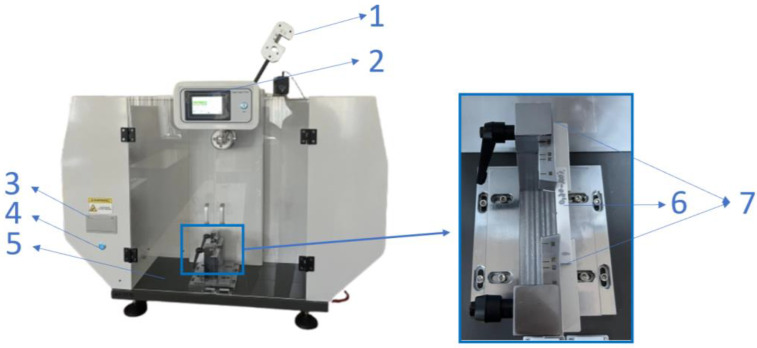
Impact testing machine HST XJJD-50T: 1—7.5J hammer; 2—display; 3—printer; 4—ON/OFF button; 5—safety doors; 6—impact sample; 7—adjustable spans.

All 90 samples additively manufactured by the QIDI Q1 Pro 3D printer were impact tested (Charpy method) on HST XJJD-50T equipment (Jinan, China), (Figure 7) using a 7.5J hammer and an impact velocity of 2.9 m/s.

**Figure 8:**
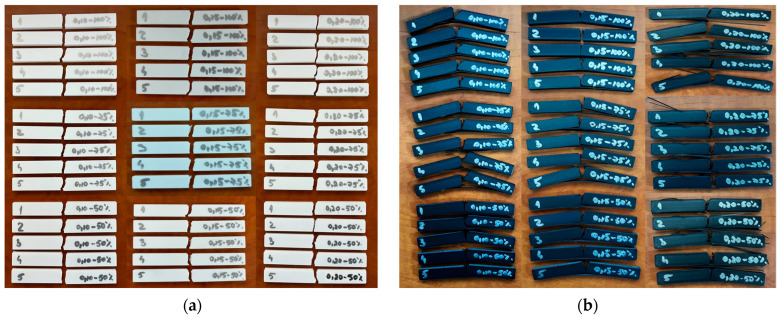
Additively manufactured samples from the QIDI Q1 Pro printer after impact testing on the HST XJJD-50T machine: (**a**) PETG; (**b**) rPETG.

Figure 8 shows the 90 impact samples after carrying out experimental impact determinations with the HST XJJD-50T equipment.

### 2.5. Experimental Design and Statistical Analysis

Using a design of experiments (DOE) analysis, the influence of the thermoplastic extrusion process parameters on the impact strengths of additively manufactured PETG and rPETG samples was evaluated. To achieve this goal, a full factorial design analysis (3^2^) was performed based on the values of the variable thermoplastic extrusion parameters L_h_ = (0.10; 0.15; 0.20) mm and I_d_ = (50; 75; 100)%. For the accuracy of the results and the experimental validation of this study, all 9 experimental conditions were replicated 5 times.

The impact strengths of additively manufactured PETG and rPETG samples were determined experimentally, and the influence of the thermoplastic extrusion process parameters (L_h_ and I_d_) was statistically evaluated using analysis of variance (ANOVA) and Minitab 20.3 software. By performing this analysis, the effects and interactions of the variable thermoplastic extrusion parameters L_h_ = (0.10; 0.15; 0.20) mm and I_d_ = (50; 75; 100)% on the impact strengths were quantified.

## 3. Results and Discussion

Table 2 and Table 3 summarize the impact resistance values for the samples that were additively manufactured using the QIDI Q1 Pro 3D printer (see Figure 5). The samples were fabricated through thermoplastic extrusion of PETG and rPETG filaments, obtained following mechanical tests performed using the HST XJJD-50T machine.

To determine the impact resistance, the relation presented below was used:

(1) $$K=\frac{E}{b\times h}\;\times 10^{3\;}\left(\frac{\mathrm{kJ}}{m^{2}}\right),$$

where *E* represents the impact energy (*J*), *b* is the specimen width (mm) and *h* is the specimen height (mm).

**Figure 9:**
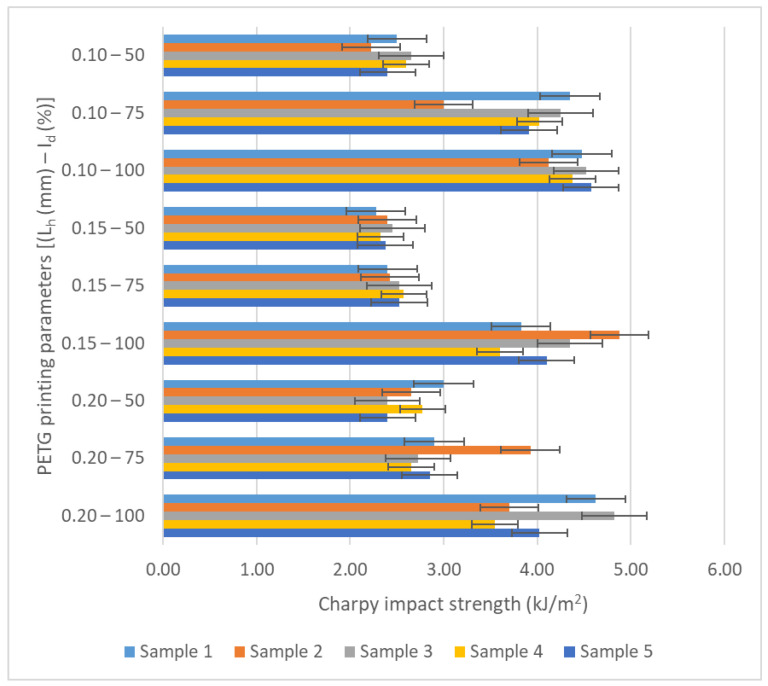
Graphical representation of impact resistance values of PETG sampes additively fabricated through thermoplastic extrusion.

**Figure 10:**
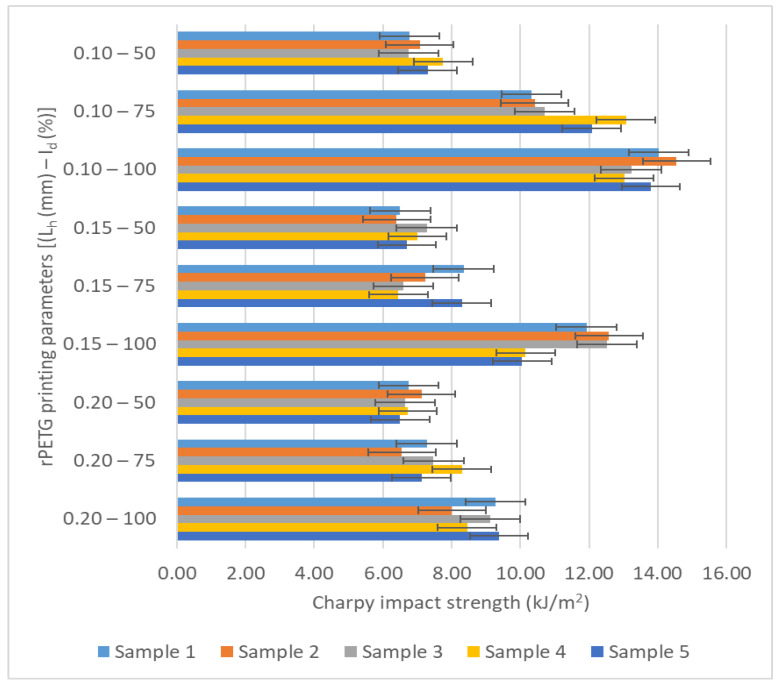
Graphical representation of impact resistance values of rPETG sampes additively fabricated through thermoplastic extrusion.

Figure 9 and Figure 10 graphically represent the impact resistance values for samples that were additively manufactured using the QIDI Q1 Pro 3D printer (see Figure 5). The samples were fabricated by thermoplastic extrusion of PETG and rPETG filaments, and subsequently the impact resistances were obtained following mechanical tests performed by the HST XJJD-50T machine (see Figure 7).

Based on the data presented in Table 2 and graphs from Figure 9, it can be observed how the variable parameters of thermoplastic extrusion, the layer height (L_h_) and the filling percentage (I_d_), influenced the impact strengths of the samples additively manufactured by thermoplastic extrusion of PETG. The highest values of the average impact strengths (4.03–4.58) kJ/m2 were obtained for the PETG samples manufactured with I_d_ = 100%. The maximum average impact strength (4.58 kJ/m^2^) was achieved for the PETG samples manufactured with L_h_ = 0.10 mm and I_d_ = 100%. Increasing the filling percentage of PETG samples from 50% to 75% increased their impact resistance by (5.29–47.71)%, and increasing the filling percentage of PETG samples from 75% to 100% generated an increase in their impact resistance by (13–66.47)%.

Based on the data presented in Table 3 and the graphs from Figure 10, the influence of the variable parameters of thermoplastic extrusion, the height of the layer (L_h_) and the filling percentage (I_d_), on the impact resistance of the samples manufactured by additive manufacturing by thermoplastic extrusion of rPETG can be observed. The highest values of the average impact resistance (8.85–13.73) kJ/m^2^ were obtained for the rPETG samples manufactured with I_d_ = 100%. The maximum average impact resistance (13.73 kJ/m^2^) was achieved for the rPETG samples manufactured with L_h_ = 0.10 mm and I_d_ = 100%. Increasing the filling percentage of rPETG samples from 50% to 75% led to an increase in their impact resistance by (8.10–37.01)%, and increasing the filling percentage of rPETG samples from 75% to 100% generated an increase in their impact resistance by (16.96–35.47)%.

**Figure 11:**
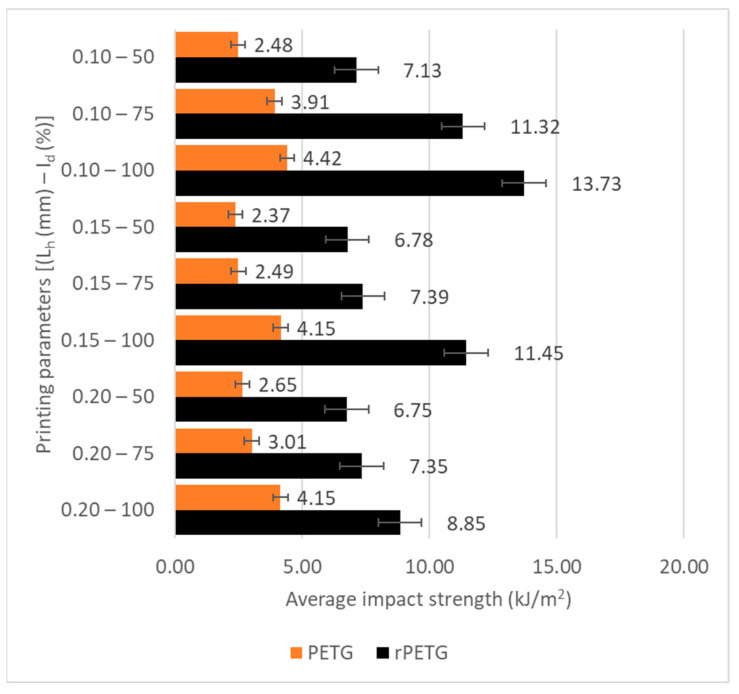
Average impact strength values for samples additively manufactured by thermoplastic extrusion of PETG and rPETG.

Figure 11 graphically represents the average impact resistance values for the samples additively fabricated by the QIDI Q1 Pro printer (see Figure 5), by thermoplastic extrusion of PETG and rPETG filaments, obtained following mechanical tests performed using the HST XJJD-50T machine (see Figure 7).

According to Figure 11 it can be concluded that the samples manufactured additively by thermoplastic extrusion of rPETG had higher impact strengths than those of the samples made of PETG, regardless of the values of the variable parameters. The overall average of the impact strengths calculated for the 45 samples manufactured additively by thermoplastic extrusion of rPETG was higher by 5.68 kJ/m^2^ (172.62%) compared with the overall average of the impact strengths calculated for the 45 samples manufactured additively by thermoplastic extrusion of PETG. For L_h_ = (0.10; 0.15; 0.20) mm and I_d_ = 50%, the impact strengths of the samples manufactured additively by thermoplastic extrusion of rPETG were higher by (169.57–185.41)% compared with the impact strengths of the samples manufactured additively by thermoplastic extrusion of PETG. For L_h_ = (0.10; 0.15; 0.20) mm and I_d_ = 75%, the impact strengths of the samples additively manufactured by thermoplastic extrusion of rPETG were higher by (189.74–194.98)% compared with the impact strengths of the samples additively manufactured by thermoplastic extrusion of PETG. For L_h_ = (0.10; 0.15; 0.20) mm and I_d_ = 100%, the impact strengths of the samples additively manufactured by thermoplastic extrusion of rPETG were higher by (113.39–210.87)% compared with the impact strengths of the samples additively fabricated by thermoplastic extrusion of PETG.

## 4. Statistical Evaluation of the Influence of FDM Parameters on Impact Resistance and Their Optimization

To investigate the influence of the variable parameters of thermoplastic extrusion, the height of the layer (L_h_) and the filling percentage (I_d_), on the impact resistance of the additively manufactured samples from PETG and rPETG, Minitab 20.3 software and the following analyses were used: ANOVA (analysis of variance), DOE (design of experiments), response optimizer and regression [54].

**Figure 12:**
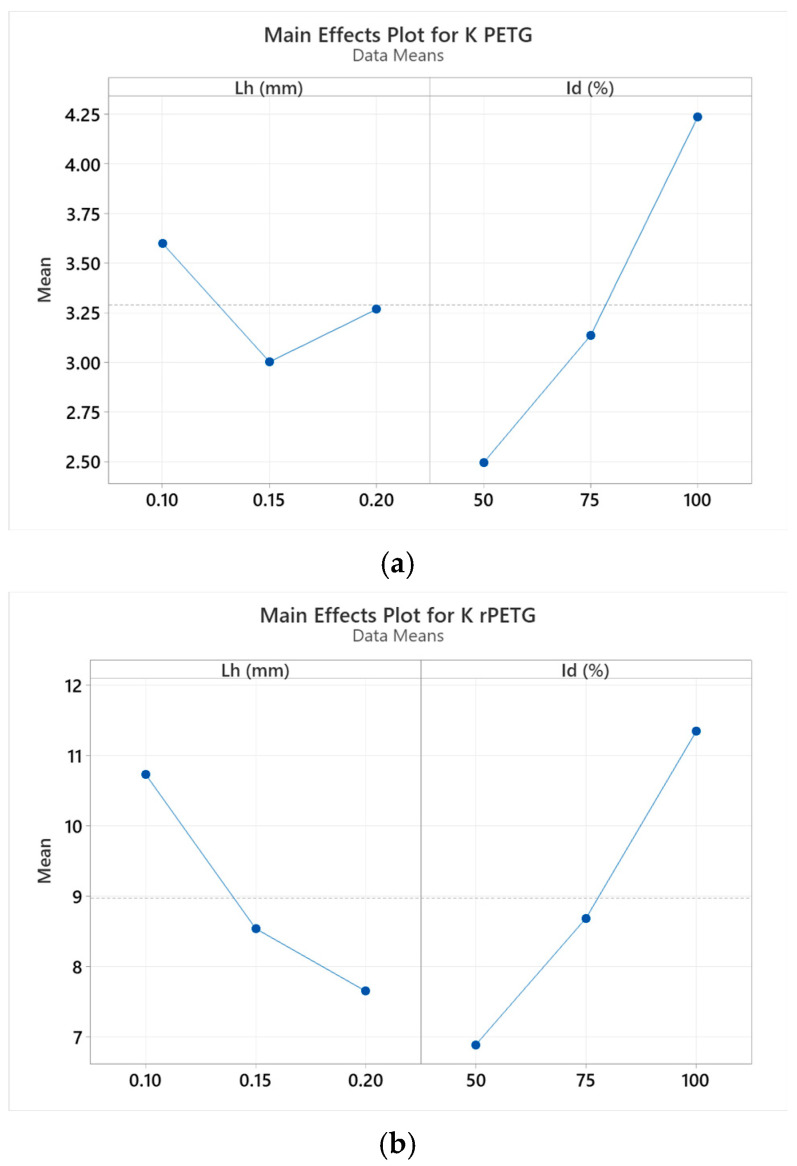
Main effects plots of factors influencing impact resistances for (**a**) PETG; (**b**) rPETG.

Figure 12 presents graphs representing the influence of the variable parameters of thermoplastic extrusion, L_h_ = (0.10; 0.15; 0.20) mm and I_d_ = (50; 75; 100)%, on the impact strengths (K) of additively manufactured samples from PETG (Figure 12a) and rPETG (Figure 12b).

The analysis of thermoplastic extrusion parameters in relation to the impact strength (K) of PETG samples (Figure 12a) highlighted that the filling percentage (I_d_) was the dominant factor influencing impact resistance. The impact strength values increased significantly with an increase in filling percentage. For I_d_ = 50% the impact strength value was 2.48 kJ/m^2^. By increasing the filling percentage to 100% the impact strength value increased by 78.23%. Figure 12b highlights the influence of both variable thermoplastic extrusion parameters on the impact strengths (K) of rPETG samples.

**Figure 13:**
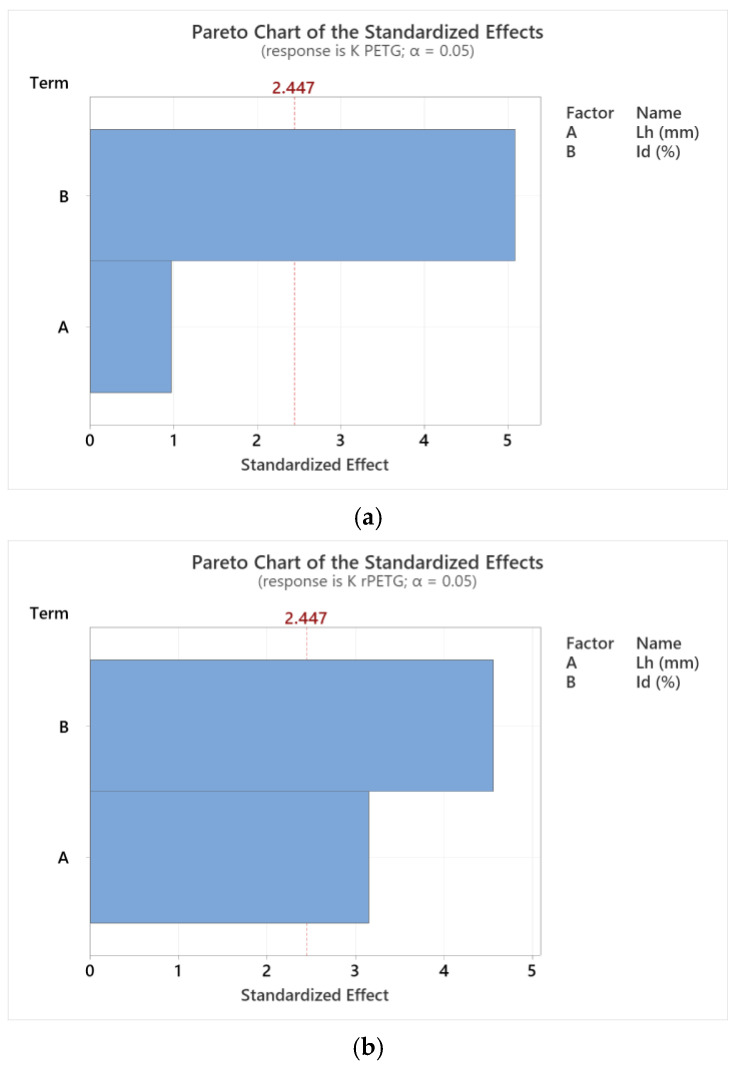
Pareto charts illustrating the influence of variable thermoplastic extrusion parameters (A = L_h_ and B = I_d_) on impact strengths for (**a**) PETG; (**b**) rPETG.

Using the variable thermoplastic extrusion parameters presented in Table 1 [L_h_ = 0.10; 0.15; 0.20) mm and I_d_ = (50; 75; 100)%], the average results of the impact strengths from Table 2 and Table 3 and the Minitab 20.3 software are presented by the Pareto graphs in Figure 13. These graphs express the influence of the variable parameters on the impact strengths of the samples manufactured from PETG and rPETG.

The Pareto charts presented in Figure 13 express the influence of the variable parameters of thermoplastic extrusion (A = L_h_ and B = I_d_) on the impact resistance of additively manufactured samples made from PETG (Figure 13a) and rPETG (Figure 13b). According to Figure 13a, the factor B = I_d_ significantly influenced the impact strengths of the samples additively manufactured by thermoplastic extrusion of PETG, the influence of the factor A = I_d_ being not statistically insignificant. According to Figure 13b, both parameters of thermoplastic extrusion (A = L_h_ and B = I_d_) influenced the impact strengths of the samples made with rPETG, factor B = I_d_ being the factor that had a 46.67% greater influence compared with factor A = L_h_.

**Figure 14:**
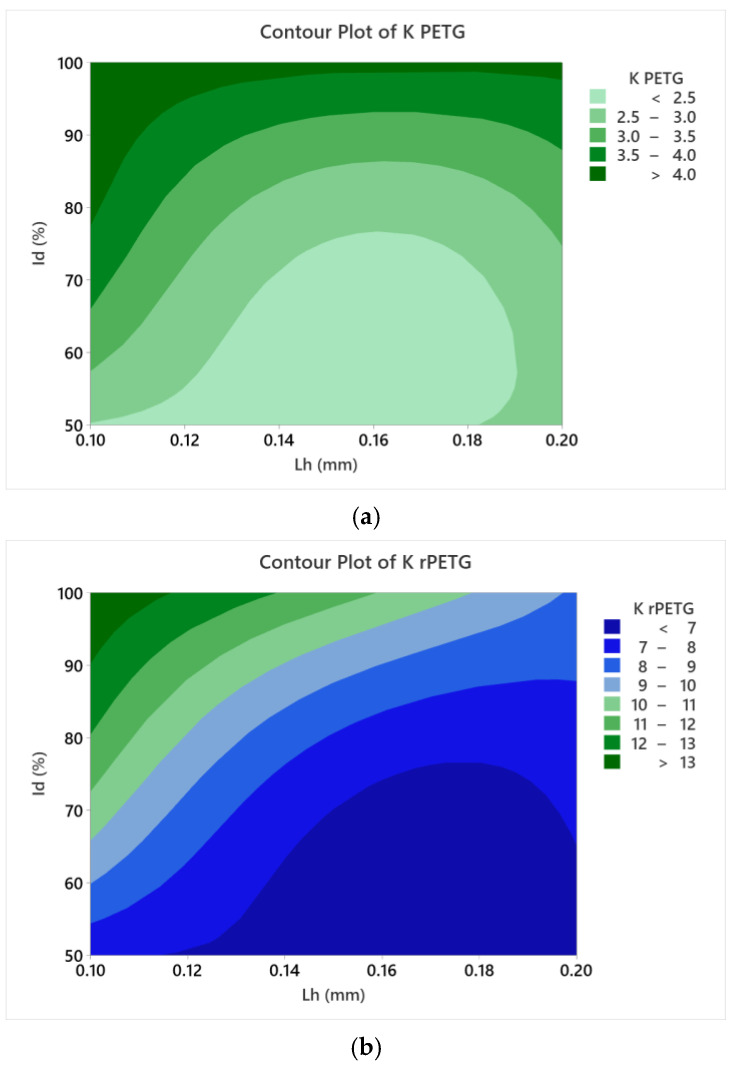
Influence of FDM process variables parameters (L_h_ and I_d_) on impact resistance, represented through contour plots for (**a**) PETG; (**b**) rPETG.

Contour plots presented in Figure 14 were generated in Minitab 20.3 using the thermoplastic extrusion parameters and corresponding impact strength values presented in Table 2 and Table 3.

The contour plots in Figure 14 show how the thermoplastic extrusion parameters (L_h_ and I_d_) affect the impact strengths of additively manufactured PETG (Figure 14a) and rPETG (Figure 14b) samples. According to Figure 14, the impact strength (K) increased when the filling percentage (I_d_) increased and the layer height deposited per pass (L_h_) decreased.

**Figure 15:**
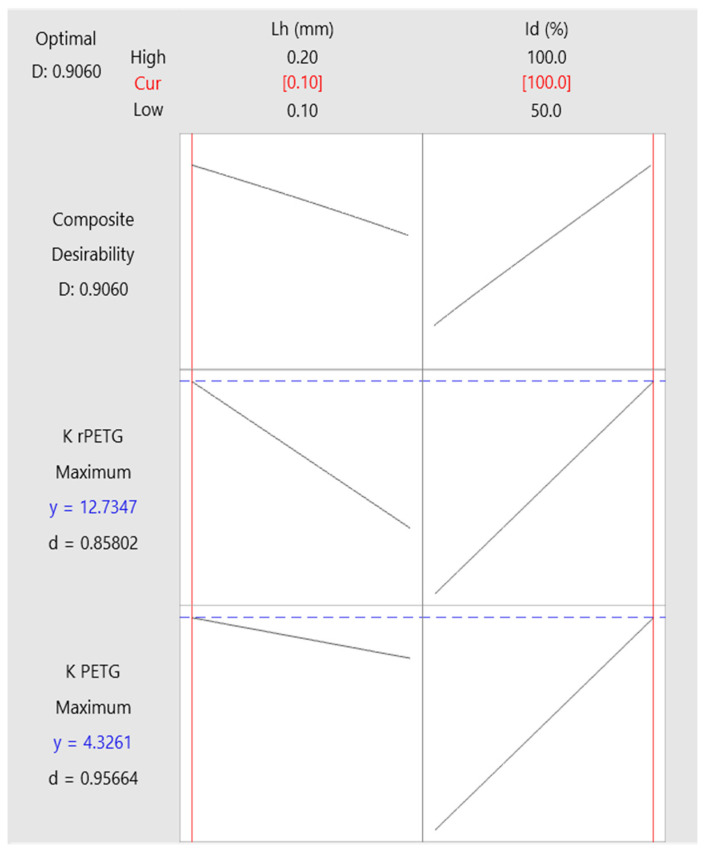
Parameter optimization graphs for additive manufacturing by thermoplastic extrusion of PETG and rPETG impact samples.

The optimization of printing parameters plays an essential role in the efficient use of the 3D printer and on the mechanical characteristics of the additively manufactured parts. With the objective function of increasing the impact resistance values of the additively manufactured PETG and rPETG samples, the variable parameters of the thermoplastic extrusion were optimized, the layer height deposited per pass L_h_ = (0.10; 0.15; 0.20) mm and the filling percentage I_d_ = (50; 75; 100)%. Figure 15 presents the results of the multi-objective optimization to determine the optimal parameters of thermoplastic extrusion for the additive manufacturing of parts made of PETG and rPETG filament. To achieve the optimization, the variable parameters of thermoplastic extrusion were used (the height of the deposited layer L_h_ = (0.10; 0.15; 0.20) mm and the filling percentage I_d_ = (50; 75; 100)%) and the objective functions consisted of maximizing the impact resistances (K) of the samples manufactured additively from PETG and rPETG filament. To achieve the optimization, the composite desirability function was used, the value of which was D = 0.9060, indicating that the proposed solution represents an effective choice for both types of materials.

In the graphs in Figure 15, the black lines represent the direction and intensity of the influence of the factors (L_h_ and I_d_) on the responses (K), the red vertical lines represent the optimal values of the factors and the blue horizontal lines indicate the maximum values of the responses. The optimal configuration indentified was a layer height (L_h_) of 0.10 mm and an infill percentage (I_d_) of 100%.

Using Minitab 20.3 software, the average impact strength values from Table 2 and Table 3 and the variable parameters of thermoplastic extrusion (I_d_ and L_h_), regression equations were obtained that predicted the impact strength values for each material studied.

(2) $$K\;PETG=3.289-0.310\cdot L_{h}\left(0.10\right)\;\mathrm{mm}-0.287\cdot L_{h}\left(0.15\right)\;\mathrm{mm}-0.022\cdot \;L_{h}\left(0.20\right)\;\mathrm{mm}-0.794\cdot I_{d}\left(50\right)\%-0.153\cdot I_{d}\left(75\right)\%+0.948\cdot I_{d}\left(100\right)\%$$

(3) $$K\;rPETG=8.969+1.756\cdot L_{h}\left(0.10\right)\;\mathrm{mm}-0.434\cdot L_{h}\left(0.15\right)\;\mathrm{mm}-1.322\cdot \;L_{h}\left(0.20\right)\;\mathrm{mm}-2.084\cdot I_{d}\left(50\right)\%-0.286\cdot I_{d}\left(75\right)\%+2.369\cdot I_{d}\left(100\right)\%$$

## 5. Conclusions

This paper presents the results of this study on the evaluation of the influence of thermoplastic extrusion parameters on the impact strengths (K) of samples additively manufactured from PETG and recycled PETG (rPETG) filament from the Everfil brand. In this context, 90 impact samples, 45 from PETG and 45 from rPETG, were additively manufactured by thermoplastic extrusion by the QIDI Q1 Pro printer, with the layer height deposited per pass L_h_ = 0.10/0.15/0.20 mm and the filling percentage I_d_ = 50/75/100%, which were subsequently subjected to impact testing by the HST XJJD-50T machine, using the 7.5J hammer and the impact speed of 2.9 m/s.

The key conclusions are as follows:

- The considered variable parameters, L_h_ = (0.10; 0.15; 0.20) mm and I_d_ = (50, 75, 100)%, influence the impact strengths (K) of PETG and rPETG samples (see Figure 12, Figure 13 and Figure 14);
- The general average of the impact strengths corresponding to the samples manufactured from rPETG is higher by 172.64% compared with the general average of the impact strengths corresponding to the samples manufactured from PETG;
- The filling percentage (I_d_) is the variable parameter that significantly affects the impact strengths (K);
- The optimal parameters for additive manufacturing by thermoplastic extrusion of PETG and rPETG impact samples are L_h_ = 0.10 mm and I_d_ = 100% (see Figure 15);
- For L_h_ = 0.10 mm and I_d_ = 100%, the impact strengths of the samples manufactured additively by thermoplastic extrusion of rPETG are 210.87% higher than the impact strengths of samples manufactured additively by thermoplastic extrusion of PETG.

The results obtained show that decreasing the height of the deposited (L_h_) generates an increase in the adhesion between the layers, leading to an increase in the impact resistance of the samples manufactured by additively manufactured thermoplastic extrusion of PETG and rPETG. Increasing the filling percentage (I_d_) determines an increase in the impact resistance due to the decrease in porosity, internal defects and the increase in the adhesion between the layers, this being also highlighted in works [37,38,41,42].

Considering the results obtained from this study and others undertaken by the same authors, we can conclude that the use of rPETG in the field of additive manufacturing technologies offers multiple advantages compared with the use of virgin PETG, e.g., superior mechanical characteristics [26,27,43], reduction in costs corresponding to the acquisition of filament [55] and reduction in negative effects on the environment [56].

This study provides recommendations on setting 3D printing parameters to obtain optimal mechanical characteristics, as well as a comparative analysis of the mechanical performances corresponding to parts manufactured by thermoplastic extrusion of PETG and rPETG, thus certifying the viability of using recycled materials in the field of additive manufacturing technologies.

Future directions of study include performing microstructural analysis by scanning electron microscopy (SEM) to investigate the structure of the parts.

## Figures and Tables

**Table 1:** FDM printing parameters used to manufacture impact samples from PETG and rPETG.

Thermoplastic Extrusion 3D Printing Parameters	QIDI Q1 PRO
Materials, Mat	PETG; rPETG
Part orientation, P_o_	XY
Extruder temperature, E_t_ Platform temperature, P_t_ Printing speed, P_s_ First layer speed, F_ls_	250 °C 70 °C 120 mm/s 50 mm/s
Plate adhesion, P_a_	Brim
Layer height, L_h_	0.10; 0.15; 0.20 mm
First layer height, F_lh_	0.30 mm
Top layers, T_l_	5
Bottom layers, B_l_	3
Infill percentage, I_d_	50; 75; 100%
Infill structure, I_s_	Rectilinear
Infill angle, I_a_	45°
Top fill pattern, T_f_	Monotonic lines
Bottom fill pattern, B_f_	Monotonic lines
Nozzle diameter, N_d_	0.40 mm

**Table 2:** Impact strength results for samples additively manufactured by thermoplsastic extrusion of PETG.

Layer Height, L_h_	Infill Percentage, I_d_	Charpy Impact Strength, K (kJ/m^2^)					Average
(mm)	(%)	Sample Number					(kJ/m^2^)
		1	2	3	4	5	
0.10	50	2.50	2.23	2.65	2.60	2.40	2.48
	75	4.35	3.00	4.25	4.03	3.91	3.91
	100	4.48	4.13	4.53	4.38	4.58	4.42
0.15	50	2.28	2.40	2.45	2.33	2.38	2.37
	75	2.40	2.43	2.53	2.58	2.53	2.49
	100	3.83	4.88	4.35	3.60	4.10	4.15
0.20	50	3.00	2.65	2.40	2.78	2.40	2.65
	75	2.90	3.93	2.73	2.65	2.85	3.01
	100	4.63	3.70	4.83	3.55	4.03	4.15

**Table 3:** Impact strength results for samples additively manufactured by thermoplastic extrusion of rPETG.

Layer Height, L_h_	Infill Percentage, I_d_	Charpy Impact Strength, K (kJ/m^2^)					Average
(mm)	(%)	Sample Number					(kJ/m^2^)
		1	2	3	4	5	
0.10	50	6.78	7.08	6.75	7.75	7.30	7.13
	75	10.33	10.43	10.70	13.08	12.08	11.32
	100	14.03	14.55	13.23	13.03	13.80	13.73
0.15	50	6.50	6.40	7.28	7.00	6.70	6.78
	75	8.35	7.23	6.60	6.45	8.30	7.39
	100	11.93	12.58	12.53	10.15	10.05	11.45
0.20	50	6.75	7.13	6.65	6.73	6.50	6.75
	75	7.28	6.55	7.48	8.30	7.13	7.35
	100	9.28	8.00	9.13	8.45	9.38	8.85

## Data Availability

Data are contained within the article. The data presented in this study are available on request from the corresponding.
